# Revision of *Macroponema* Mawson, 1978 (Nematoda: Strongylida) from macropodid marsupials with the description of two new species

**DOI:** 10.1186/s13071-020-04129-8

**Published:** 2020-06-10

**Authors:** Tanapan Sukee, Abdul Jabbar, Ian Beveridge

**Affiliations:** Department of Veterinary Biosciences, Melbourne Veterinary School, The University of Melbourne, Werribee, VIC 3030 Australia

**Keywords:** *Macroponema*, Strongyloidea, Marsupials, Australia, New species

## Abstract

**Background:**

Species of *Macroponema* Mawson, 1978 are strongyloid nematodes which occur in the stomachs of macropodid marsupials in Australia. In this study, the genus *Macroponema* is revised, redescriptions of the two known species are provided, and two new species are added to the genus.

**Methods:**

A molecular characterisation of the internal transcribed spacers of the nuclear ribosomal DNA of representative specimens of *Macroponema* from all known host species was undertaken to confirm the status of *M*. cf. *comani*. This resulted in the identification of a further new species within the genus. Consequently, a review of all available material in museum collections was undertaken.

**Results:**

The two known species *M. beveridgei* Mawson, 1978 from *Osphranter antilopinus* (Gould) and *O. robustus* (Gould), and *M. comani* Mawson, 1978 from *Macropus giganteus* Shaw are re-described and their geographical distributions expanded. Two new species added to the genus are *M. arundeli* n. sp. from *Ma. giganteus* found in Queensland and the north east of New South Wales, and *M. obendorfi* n. sp. from *O. antilopinus* and *O. robustus* in the Northern Territory, the Kimberley Division of Western Australia and eastern Queensland. The latter species was formerly identified as *M*. cf. *comani* based on molecular studies. The specific identification of both of the new species is supported by ribosomal DNA sequence data.

**Conclusions:**

Based on the morphological and molecular characterisation of nematodes, this study has revealed the existence of four species within the genus *Macroponema*. The current phylogenetic data suggest that *Macroponema* spp. plausibly evolved by host switching; however, further studies are required to test this hypothesis.
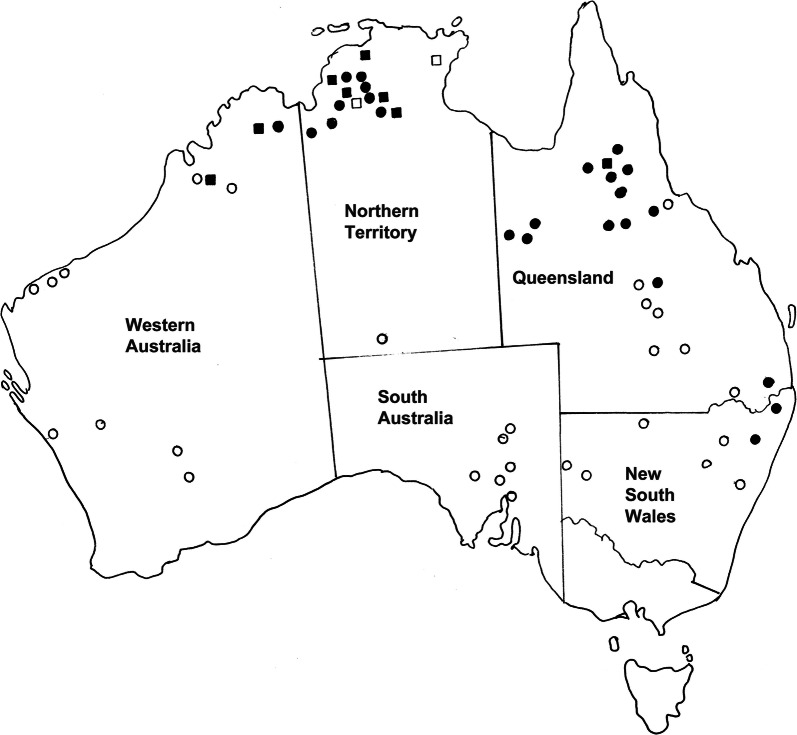

## Background

*Macroponema* Mawson, 1978 was established by Mawson [1] to accommodate two new species of strongyloid nematodes, *M. beveridgei* Mawson, 1978 and *M. comani* Mawson, 1978, from the stomachs of macropodid marsupials in eastern and northern Australia. Initially placed in the family Trichonematidae Wittenberg, 1925, by Mawson [1], the genus was placed by Lichtenfels [2] within the newly erected tribe Macropostrongylinea Lichtenfels, 1980 in the Cloacininae (Stossich, 1899), along with the other genera of nematodes found in the stomachs of macropodid marsupials, a taxonomic position subsequently upheld by Gibbons [3]. More recently, Tan et al. [4] reported specimens morphologically similar to *M. comani*, but occurring in northern wallaroos, *Osphranter robustus woodwardi* (Thomas), rather than in the usual host, *Macropus giganteus* Shaw and based on differences in sequences of the internal transcribed spacers of the nuclear ribosomal DNA suggested that their specimens represented a cryptic species within *M. comani*, designated as *M*. cf *comani*.

In the present study, a molecular examination of representative specimens of *Macroponema* from all known host species was undertaken to confirm the status of *M*. cf *comani*. However, this resulted in the identification of a further new species within the genus. Consequently, a review of all available material in museum collections was undertaken, resulting in re-descriptions of the two known species, the description of *M*. cf *comani* as a new species as well as the description of another new species.

## Methods

### Collection of samples

Specimens of nematodes were collected opportunistically from: (i) fresh, road-killed kangaroos; (ii) kangaroos killed by commercial shooters; (iii) kangaroo culls; or (iv) animals used in related parasitological studies. In some instances, entire gastrointestinal tracts were preserved in formalin; in other instances, stomach contents were preserved in either formalin or 70% ethanol. Nematodes retrieved from these sources were stored in 70% ethanol. When fresh material was available, samples for morphological analysis were preserved in formalin or ethanol, while those required for molecular analysis were either frozen immediately in liquid nitrogen or were fixed in 70% ethanol and all were stored at − 80 °C until use.

### Morphological studies

The anterior and posterior extremities of nematodes were excised and cleared in lactophenol for identification while the mid-section of the body was stored in 70% ethanol for molecular studies. The anterior and posterior extremities of specimens used for molecular studies were then stored in 70% ethanol and deposited in the Australian Helminthological Collection (AHC) of the South Australian Museum, Adelaide (SAM). Collection localities are listed under states with individual localities listed in order of increasing latitude (Table 1).

**Table 1:** Specimens of *Macroponema* included in the molecular analysis

Species	Host	Locality	Code	GenBank ID	SAM ID
*Macroponema arundeli* n. sp.	*Macropus giganteus*	50 km N of The Lynd Junction, Qld	50Y2-3	MT080008-9	48906
*Macroponema arundeli* n. sp.	*Macropus giganteus*	Prairie, Qld	22F5	MT080011	48922
*Macroponema arundeli* n. sp.	*Macropus giganteus*	Kelso, Townsville, Qld	25W2	MT080010	48923
*Macroponema arundeli* n. sp.	*Macropus giganteus*	Bogantungen, Qld	1W6	MT080012	48920
*Macroponema beveridgei*	*Osphranter robustus*	Charters Towers, Qld	RG5	HE775534	35117
*Macroponema beveridgei*	*Osphranter robustus*	74 km W of Cloncurry, Qld	21P4	MT080016	48921
*Macroponema beveridgei*	*Osphranter robustus*	Devonport Stn via Cloncurry, Qld	21W2	MT080015	48927
*Macroponema beveridgei*	*Osphranter robustus*	Prairie, Qld	22D1	MT080014	48891
*Macroponema beveridgei*	*Osphranter robustus*	18 km W of Warwick, Qld	7M53-4	MT080018-9	48926
*Macroponema beveridgei*	*Osphranter robustus*	27 km E of Mt Surprise, Qld	8B5	MT080017	48928
*Macroponema beveridgei*	*Notamacropus dorsalis*	Warrawee Stn, Charters Towers, Qld	F898	HE775535	48213
*Macroponema beveridgei*	*Osphranter robustus*	Warrawee Stn, Charters Towers, Qld	AV7	MT080024	48932
*Macroponema beveridgei*	*Osphranter robustus*	Warrawee Stn, Charters Towers, Qld	AU4,8,12	MT080025-7	48917
*Macroponema beveridgei*	*Osphranter robustus*	Wollomombi, NSW	33B6	MT080013	44350^a^
*Macroponema comani*	*Macropus giganteus*	50 km N of The Lynd Junction, Qld	50Y4	MT080023	48882
*Macroponema comani*	*Macropus giganteus*	5 km S of Reid River, Qld	27R2	MT080020	48918
*Macroponema comani*	*Macropus giganteus*	35 km S of Clermont, Qld	1X6-7	MT080021-2	48925
*Macroponema comani*	*Macropus giganteus*	Darling Plains Stn via Banana, Qld	F894	HE775536	46096
*Macroponema obendorfi* n. sp.	*Osphranter robustus*	Newry Stn via Timber Creek, NT	AJ11-12	HE775532-3	46097

^a^Paragenophores deposited as vouchers in this instance

*Abbreviations*: Qld, Queensland; NSW, New South Wales; NT, Northern Territory; Stn: Station; SAM: South Australian Museum

All specimens present in the Australian Helminthological Collection of the South Australian Museum, Adelaide, were examined. Nematodes were cleared in lactophenol and examined using an Olympus BH-2 microscope. Drawings were made with a drawing tube attached to the microscope and measurements, made with an ocular micrometer are presented in millimetres as the range followed by the mean in parentheses. Type-specimens have been deposited in SAM. Higher nematode taxonomic categories follow Beveridge et al. [5]. Host nomenclature follows Jackson & Groves [6]. The geographical distribution of *M. giganteus* in maps follows van Dyck & Strahan [7]. Species descriptions are presented in alphabetical order. The known geographical distributions of the nematode species are shown, based on the host species which have been examined for parasites (Beveridge et al. unpublished data) and those in which the particular nematode species has been identified. In instances where individual localities were proximate (e.g. Stations in the vicinity of Charters Towers and the Lynd Junction in northern Queensland), these locations have been combined.

To comply with the regulations set out in Article 8.5 of the amended 2012 version of the *International Code of Zoological Nomenclature* (ICZN) [8], details of the new species have been submitted to ZooBank. The Life Science Identifier (LSID) of the article is urn:lsid:zoobank.org:pub: 8C095D38-A90D-441B-878C-83AC1D0E595C.

### Molecular and phylogenetic analyses

Genomic DNA was isolated from the mid sections of individual nematodes using the sodium-dodecyl-sulphate/proteinase K extraction method followed by column purification (Wizard Clean-up, Promega, Madison, USA). The ITS-1, 5.8S and ITS-2 regions (ITS+) within the rDNA were amplified by PCR using primers NC16 (forward; 5′-AGT TCA ATC GCA ATG GCT T-3′) and NC2 (reverse; 5′-TTA GTT TCT TTT CCT CCG CT-3′) [9]. PCRs were performed in a total volume of 50 μl containing 2 μl of DNA template, 10 mM Tris-HCl (pH 8.4), 50 mM KCl (Promega), 3.5 mM MgCl_2_, 250 μM of each deoxynucleotide triphosphate (dNTP), 100 pmol of each primer, and 1 U of GoTaq polymerase (Promega). The PCR conditions used were: 94 °C for 5 min, then 35 cycles of 94 °C for 30 s, 55 °C for 20 s, and 72 °C for 20 s, followed by 72 °C for 5 min. Negative (no DNA) and positive controls (*Paramacropostrongylus toraliformis* gDNA) were included in the assays. Amplicons was subjected to agarose gel electrophoresis (1.5% gels in 0.5 TAE buffer containing 20 mM Tris, 10 mM acetic acid, 0.5 mM EDTA) stained using GelRed Nucleic Acid Gel Stain (Biotium GelRed stain, Fisher Scientific, Waltham, Massachusetts, USA) and photographed using a gel documenting system (Kodak Gel Logic 1500 Imaging System, Eastman Kodak Company, Rochester, NY, USA).

Amplicons were purified using shrimp alkaline phosphate and exonuclease I [10] prior to automated Sanger DNA sequencing (96-capillary 3730xl DNA Analyser, Applied Biosystems, Foster City, CA, USA) at Macrogen Incorporation, South Korea. The primers NC16 and NC2 were used in separate reactions. Assessment of sequencing quality was conducted in the software Geneious Prime 2019.0.4 (http://www.geneious.com). Sequences were aligned using MUSCLE v.3.8.31 [11] and manually adjusted in the program MEGA X [12].

Phylogenetic analysis of the aligned ITS+ sequences was conducted by Bayesian inference (BI) in MrBayes [13]. The most appropriate partition scheme and the evolutionary model were determined using PartitionFinder V. 2.0 [14] under the AICc criterion. The data were partitioned into subset 1 (ITS1 and ITS2) and subset 2 (5.8S). The evolutionary models assigned were nst = 6 with a proportion of invariable sites for subset 1 and nst = 1 for subset 2. The BI analysis was conducted with the Markov chain Monte Carlo (MCMC) with three heated and one cold chain for 20 million generations sampled every 1000th generations for three runs to ensure convergence and calculate posterior probabilities (pp). At the end of each run, the standard deviation of split frequencies was < 0.01, and the PSRF (Potential Scale Reduction Factor) equals one. For each analysis, a 50%-majority rule consensus tree was constructed based on the final 75% of trees. The ITS+ sequence of *Monilonema ochetocephalum* (GenBank: HE775537) was included as the outgroup. Tree topology was visualised using the software Figtree v1.4.4 (http://tree.bio.ed.ac.uk/software/figtree/) and iTOL [15].

## Results

### Molecular characterisation

**Fig. 1:**
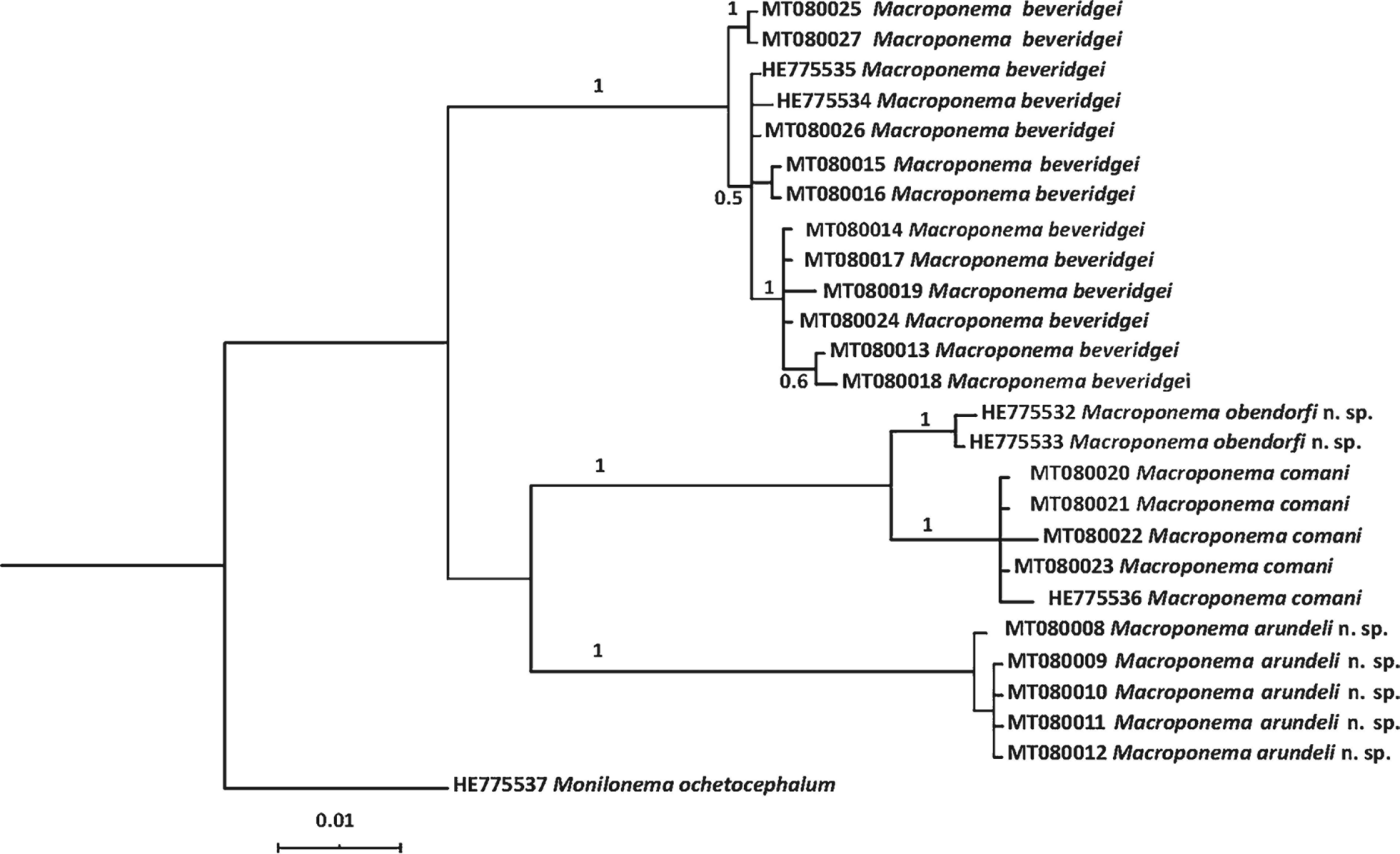
Phylogenetic associations of specimens of *Macroponema* based on a Bayesian analysis. Figures on branches represent posterior probabilities

The phylogenetic tree generated from the BI analysis included three clades (Fig. 1), each with a maximum support (pp = 1.0). One of these clades included specimens of the previously described species *M. beveridgei*. Specimens of *M. comani* were placed in a second clade and specimens previously identified as *M*. cf. *comani* by Tan et al. [4], redescribed here as *M. obendorfi* n. sp. were positioned as a sister taxon with maximum nodal support (pp = 1.0). A third clade included specimens of *M. arundeli* n. sp., which clustered with *M. comani* and *M*. cf. *comani* with limited support (pp = 0.70).

### Morphological characterisation

**Strongyloidea Weinland, 1858**

**Chabertiidae (Popova, 1955)**

**Cloacininae (Stossich, 1899)**

**Macropostrongylinea Lichtenfels, 1980**

***Macroponema*Mawson, 1978**

***Generic diagnosis***

Robust nematodes lacking alae or prominent transverse annulations; cephalic and labial collars absent; mouth dorso-ventrally elongated; 2 lateral amphids and 4 sub-median papillae, each papilla with 2 setae; buccal capsule poorly sclerotised, with anterior band of sclerotisation; buccal capsule supported externally by sets of radial muscles; oesophagus elongate, lining of corpus with sclerotised thickenings; oesophageal bulb elongate; anterior extremity of intestine enlarged; deirids in region of buccal capsule. *Male*. Bursa unornamented; ventro-ventral and ventro-lateral rays apposed; externo-lateral ray divergent; medio and postero-lateral rays apposed; externo-dorsal ray arising from lateral trunk; dorsal ray with 4 branches; external branchlets very short, recurved, terminating in elevations on internal surface of bursa; gubernaculum absent; spicules elongate, alate. *Female*. Tail conical; vulva immediately anterior to anus; vagina straight; ovejector J-shaped. *Type-species*: *Macroponema beveridgei* Mawson, 1978.

**Key to species** 
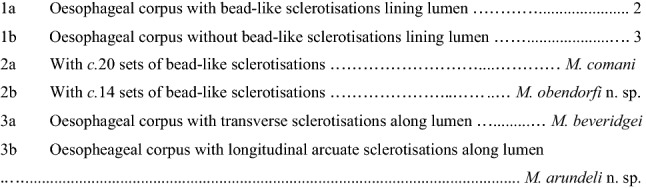


**Descriptions**

***Macroponema arundeli*Beveridge n. sp.**

***Type-host*:***Macropus giganteus* Shaw (Marsupialia: Macropodidae).

***Type-locality*:** 53 km N of The Lynd Junction (18°53′S, 144°33′E), Queensland, Australia.

***Type-material*:** Holotype ♂ (SAM AHC 48933); allotype ♀ (SAM AHC 48934); 24 paratypes: 4 ♂♂ and 20 ♀♀ (SAM AHC 48935).

***Additional material examined*:** Queensland: 2 ♂♂, 3 ♀♀, 53 km N of The Lynd Junction (SAM AHC 48907, 48909); 1 ♂, 5 ♀♀, 29 km N of The Lynd Junction (SAM AHC 48898); 1 ♂, 5♀♀, The Lynd Junction (SAM AHC 48897); 1♀, Bluewater Springs (SAM AHC 48908); 1 ♂, Woodstock (SAM AHC 48911); 4 ♂♂, 8 ♀♀, Landsdown Station via Woodstock (SAM AHC 48911); 1 ♀, 5 km S of Reid River (SAM AHC 32758); 1 ♂, 10 ♀♀, Mingela (SAM AHC 23231, 48910); 4 ♂♂, 17 ♀♀, Harvest Home Station via Charters Towers (SAM AHC 48896, 48901, 48913); 2 ♂♂, 12 ♀♀, Pallamana Station via Charters Towers (SAM AHC 48912, 48954); 6 ♂♂, 9 ♀♀, Homestead (SAM AHC 48894); 1 ♀, Prairie (SAM AHC 32592); 1 ♂, 6 km W of Prairie (SAM AHC 48904); 1 ♀, 14 km N of Clermont (SAM AHC 48900); 5 ♂♂, Inkerman Station via Home Hill (SAM AHC 48903); 1 ♂, 1 ♀, Yaamba (SAM AHC 12053); 6 ♂♂, 35 ♀♀, 66 km N of Rockhampton (SAM AHC 48895); 3 ♂♂, 1 ♀, 40 km N of Rockhampton (SAM AHC 48902); 2 ♂♂, 9 ♀♀, Rockhampton (SAM AHC 48916); 1 ♂, Darling Plains Station via Banana (SAM AHC 46096); 1 ♀, 3 km N of Miles (SAM AHC 23231); 1 ♂, 4 km east of Oman Ama (SAM AHC 48899); 3 ♂♂, 4 ♀♀, Killarney (SAM AHC 48914); New South Wales: 1 ♂, 1 ♀, Kingstown (SAM AHC 48915).

***Representative DNA sequences*:** Molecular voucher from *M. giganteus* (Prairie; SAM 48922): GenBank: MT080011 (ITS1, *5.8S* and ITS2).

***Etymology*:** Named after Dr J. H. Arundel, who first collected the specimens upon which the description of *M. comani* and the genus was based.

**Description**

***General*.** Robust, whitish nematodes. Cephalic collar absent; mouth dorso-ventrally elongate; elevation on each side of mouth opening bears lateral amphid and 2 dome-shaped sub-median papillae; papillae with 2 short, anteriorly-directed setae. Buccal capsule elongate, dorso-ventrally elongate, poorly sclerotised, with partially sclerotised annulus in anterior half; buccal capsule supported externally by strong radial musculature in posterior half; sclerotised annulus at junction of buccal capsule with oesophagus. Oesophagus elongate; corpus widest at level of and posterior to nerve-ring; posterior half of corpus with arcuate sclerotised thickenings in lining, one arising from each sector of oesophagus; isthmus narrow, elongate; bulb elongate; intestinal cells enlarged at anterior extremity, not surrounding posterior part of oesophageal bulb. Nerve-ring in anterior oesophageal region. Excretory pore at level of or posterior to oesophageal bulb; deirids at level of buccal capsule.

**Figs. 2–8:**
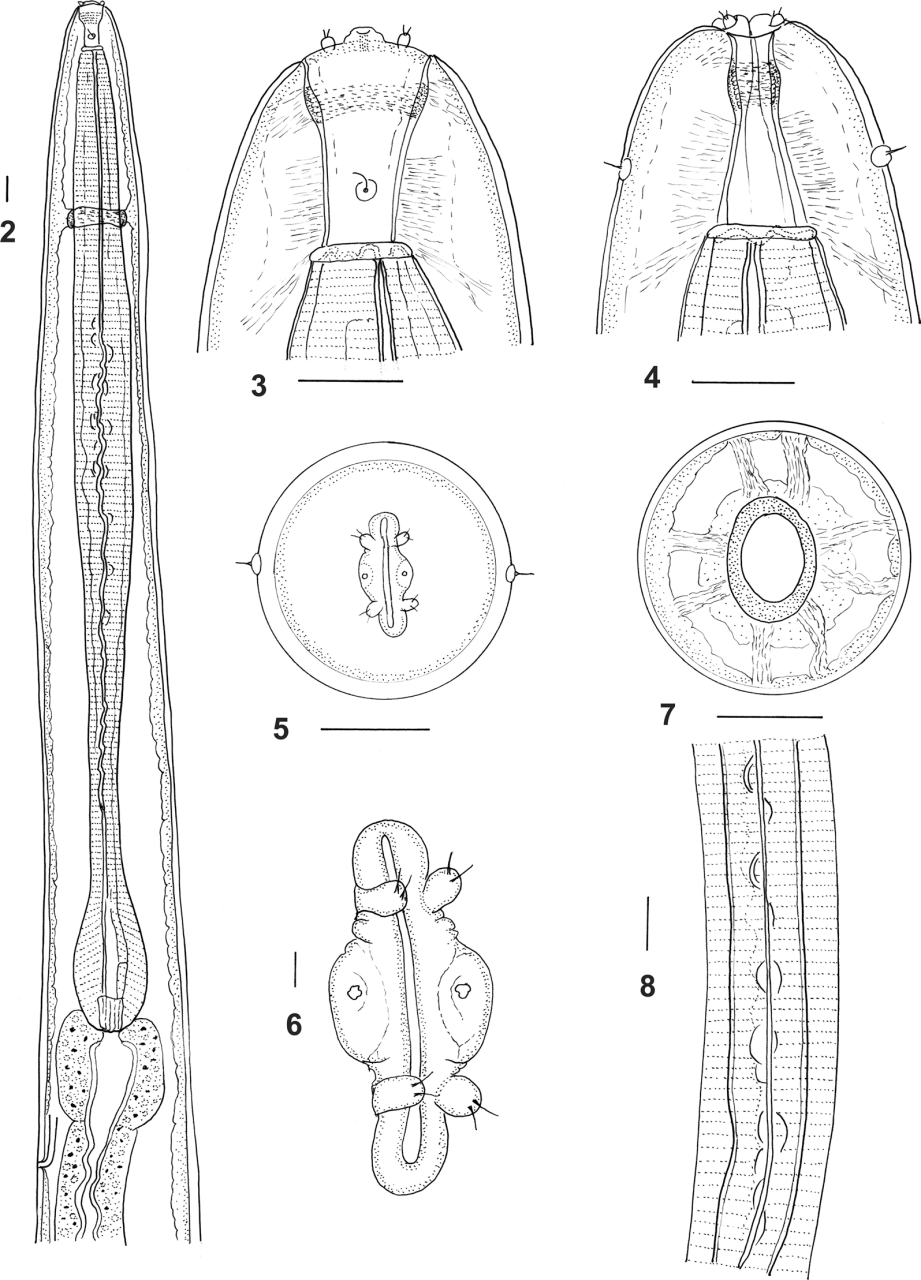
*Macroponema arundeli* n. sp. from *Macropus giganteus*. **2** Anterior region, left lateral view. **3** Buccal capsule, lateral view. **4** Buccal capsule, ventral view. **5** Anterior extremity, apical view. **6** Mouth opening, apical view, showing detail of cephalic papillae and amphids. **7** Transverse optical section through buccal capsule showing arrangement of supporting muscle bundles. **8** Oesophageal corpus, showing arcuate sclerotisation of lining. *Scale-bars*: **2**–**5**, **7**–**8**, 0.1 mm; **6**, 0.01 mm

**Figs. 9–13:**
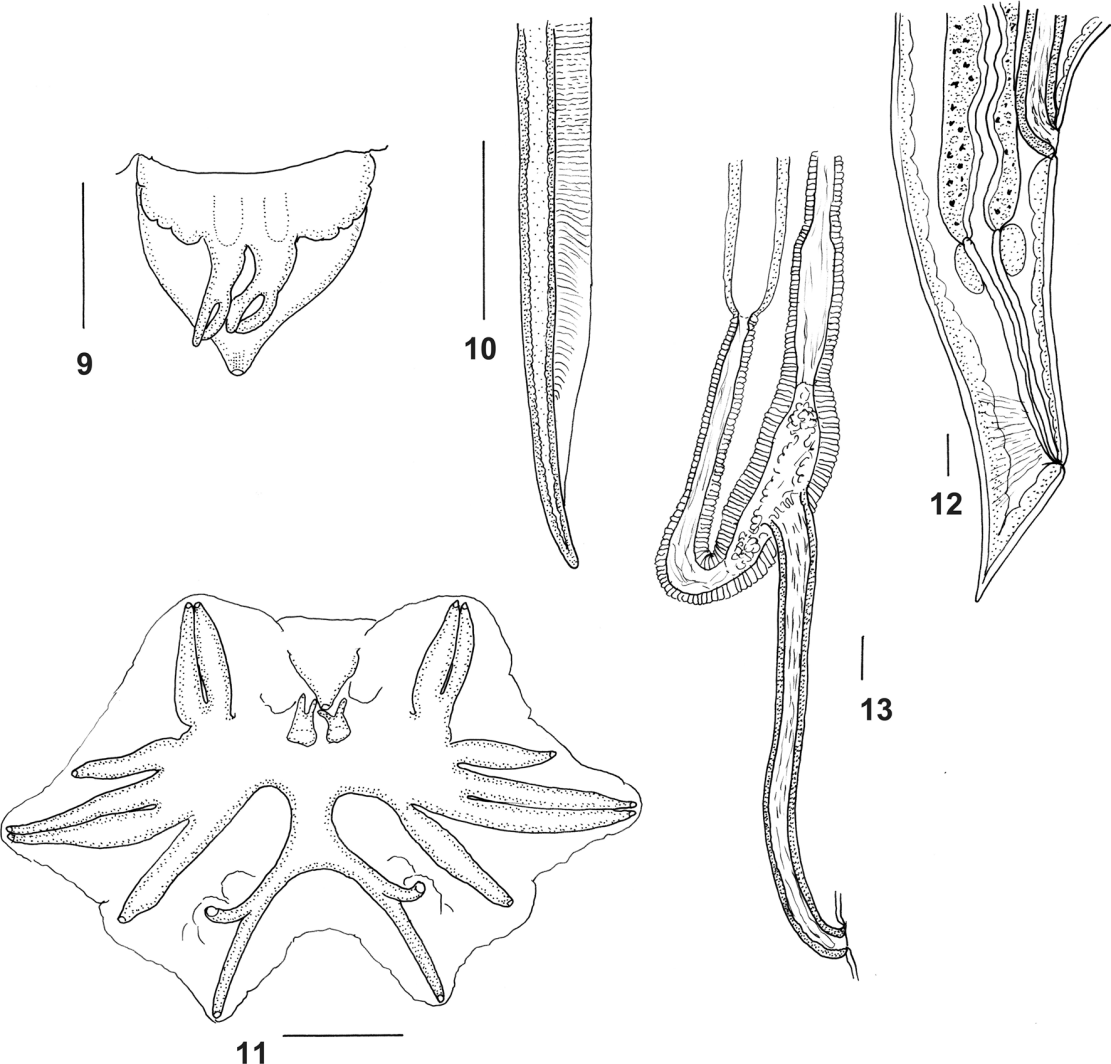
*Macroponema arundeli* n. sp. from *Macropus giganteus*. **9** Genital cone, dorsal view. **10** Spicule tip, lateral view. **11** Bursa, apical view. **12** Female tail, right lateral view. **13** Vagina and ovejector, right lateral view. *Scale-bars*: 0.1 mm

***Male*** [Measurements of 10 specimens; Figs. 2–11.] Total length 19.0–25.0 (21.7); maximum width 0.62–0.90 (0.74); buccal capsule 0.15–0.18 (0.17) long, 0.09–0.11 (0.10) wide; oesophagus 3.60–4.45 (4.13); nerve-ring from anterior extremity 0.83–1.05 (0.88); excretory pore from anterior extremity 3.50–4.68 (3.98); deirid from anterior extremity 0.11–0.14 (0.12). Bursal lobes poorly separated; lateral lobes slightly longer than ventral and dorsal lobes; dorsal lobe with median indentation; ventro-ventral and ventro-lateral rays apposed, reach margin of bursa; externo-lateral ray divergent from lateral trunk, not reaching margin of bursa; medio-lateral and postero-lateral rays apposed, reaching margin of bursa; externo-dorsal ray arising from lateral trunk, stout, not reaching margin of bursa; dorsal ray slender at origin, divides at 1/4 length; branches arcuate, internal branchlets elongate, reaching margin of bursa; external branchlets very short, arising at middle of internal branchlets, terminate in elevations on internal surface of bursa. Spicules elongate, alate; alae with numerous, fine, transverse striations; anterior extremity irregularly knobbed; distal extremity blunt-tipped; alae diminish in width gradually towards spicule tip, lose striations prior to termination; spicule length 1.95–2.40 (2.14); gubernaculum absent; central cordate and paired lateral thickenings of spicule sheaths present. Ventral lip of genital cone large, conical, bearing papilla 0; dorsal lip with paired bifid appendages.

***Female*** [Measurements of 10 specimens; Figs. 12, 13.] Total length 37.0–70.0 (49.6); maximum width 0.85–1.40 (1.13); buccal capsule 0.18–0.25 (0.20) long, 0.10–0.15 (0.12) wide; oesophagus 4.95–6.00 (5.47); nerve-ring from anterior extremity 0.93–1.25 (1.06); excretory pore from anterior extremity 4.68–6.25 (4.96); deirid from anterior extremity 0.09–0.17 (0.12). Tail short, conical, 0.38–0.53 (0.46) long, deviated slightly dorsally; vulva 1.05–1.53 (1.27) from tip of tail; vagina short, 0.90–1.30 (0.78) long, straight; eggs not seen.

**Remarks**

**Fig. 14:**
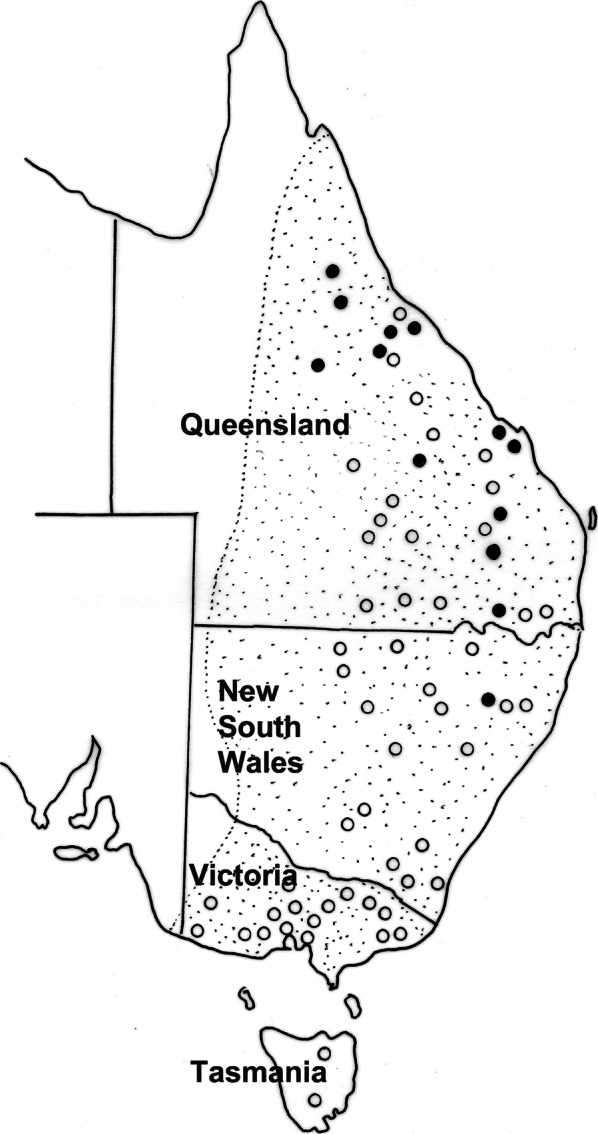
Distribution of *Macroponema arundeli* n. sp. from *Macropus giganteus* in eastern Australia. Closed circles represent localities at which the parasite has been collected; open circles indicate locations at which kangaroos have been examined but the nematode has not been found. The stippled area indicates the known geographical range of *M. giganteus*

*Macroponema arundeli* n. sp. is readily distinguishable from congeners based on the ornamentation of the oesophagus, with a unique arrangement of arcuate sclerotised formations in each dorsal and two subventral sectors of the oesophagus. This species was found only in *Ma. giganteus* in north-eastern New South Wales and Queensland (Fig. 14) and commonly co-occurred with *M. comani*. The new species can be readily distinguished from *M. comani* by its larger size, the pattern of oesophageal ornamentation, the termination of the spicular ala (abrupt in *M. comani*; gradual in the new species) and the fact that the enlarged anterior intestinal cells do not envelope the posterior region of the oesophageal bulb, as is the case in *M. comani*.

***Macroponema beveridgei*Mawson, 1978**

***Type-host*:***Osphranter antilopinus* (Gould) (Marsupialia: Macropodidae).

***Additional host*:***Osphranter robustus* (Gould) (Marsupialia: Macropodidae).

***Type-locality*:** Elizabeth Downs Station via Adelaide River (13°38′S, 130°29′E), Northern Territory, Australia.

***Type-material*:** Holotype ♂ (SAM AHC 41154); allotype ♀ (SAM AHC 41155).

***Additional material examined*:** From *Osphranter antilopinus*: Northern Territory: 6 ♂♂, 4 ♀♀, Elizabeth Downs Station via Adelaide River (SAM AHC 6083); 10 ♂♂, 10 ♀♀, Jabiluka (SAM AHC 6418); 8 ♂♂, 3 ♀♀, Marrakai Plains, 80 km SE of Darwin (SAM AHC 22992); 7 ♂♂, 44 ♀♀, Katherine (SAM AHC 32701, 34908); 1 ♀, 50 km SW of Katherine (27A2) (molecular voucher SAM AHC 49829); 9 ♂♂, 15 ♀♀, 8 km N of Mataranka (SAM AHC 34913); Western Australia: 1 ♂, 33 ♀♀, Napier Downs Station via Derby (SAM AHC 6304, 45971); 2 ♀♀, Camp Creek, Mitchell Plateau (SAM AHC 6101); Queensland: 10 ♂♂, 10 ♀♀, Burlington Station via Mount Surprise (SAM AHC 6358); 7 ♂♂, 9 ♀♀, Mount Surprise (SAM AHC 7246, 25929, 25930). From *Osphranter robustus woodwardi*: Northern Territory: 31 ♂♂, 28 ♀♀, Katherine (SAM AHC 25421, 25423, 32699, 34870, 34885, 34857); 4 ♂♂, 4 ♀♀, Mount Smith (SAM AHC 327330); 4 ♂♂, 11 ♀♀, Emerald Springs (SAM AHC 34905); 6 ♂♂, 46 ♀♀, Willeroo Station via Katherine (SAM AHC 34883); 7 ♂♂, 35 ♀♀, 5 km N of Mataranka (SAM AHC 34868); 11 ♂♂, 5 ♀♀, Pine Creek (SAM AHC 34902); 2 ♂♂, 10 ♀♀, Victoria River (SAM AHC 34891); 2 ♀♀, Newry Station via Timber Creek (SAM AHC 34893); Western Australia: 1 ♀, Mabel Downs Station via Kununurra (SAM AHC 48892). From *Osphranter robustus erubescens* (Sclater): Queensland: 28 ♂♂, 23 ♀♀, Oban Station via Mount Isa (SAM AHC 11654-6); 11 ♂♂, 28 ♀♀, Devoncourt Station via Cloncurry (SAM AHC 31708, 32518, 32560, 48890); 5 ♂♂, 27 ♀♀, Cloncurry (SAM AHC 32387, 32410, 32420, 32428). From *Osphranter robustus robustus* (Gould): Queensland: 4 ♂♂, 3 ♀♀, 24 km E of Georgetown (SAM AHC 25425): 3 ♂♂, 7 ♀♀, 17 km E of Mount Surprise (SAM AHC 25426); 2 ♂♂, 2 ♀♀, Whitewater Station via Mount Surprise (SAM AHC 25424); 2 ♀♀, 34 km N of The Lynd Junction (SAM AHC 48887); 2 ♀♀, 27 km N of The Lynd Junction (SAM AHC 48886); 1 ♂, 5 ♀♀, 8 km N of The Lynd Junction (SAM AHC 48330); 1 ♀, Kangaroo Hills Station via Greenvale (SAM AHC 32744); 1 ♀, Bluewater Springs (SAM AHC 25422); 1 ♂, 2 ♀♀, Clarke River Station via Charters Towers (SAM AHC 48888); 2 ♀♀, 74 km N of Charters Towers (SAM AHC 48885); 2 ♀♀, 60 km N of Charters Towers (SAM AHC 48889); 14 ♂♂, 5 ♀♀, Hillgrove Station via Charters Towers (SAM AHC 32534); 10 ♂♂, 10 ♀♀, Fletcher View Station via Charters Towers (SAM AHC 7411); 17♂♂, 6 ♀♀, Charters Towers (SAM AHC 7634, 7660); 5 ♂♂, 9 ♀♀, Warrawee Station via Charters Towers (SAM AHC 12041, 12320, 25428); 54 ♂♂, 12 ♀♀, Harvest Home Station via Charters Towers (SAM AHC 13519, 13520, 13521, 15610, 19871, 19872); 5 ♂♂, 11 ♀♀, Prairie (SAM AHC 32572); 10 ♂♂, 38 ♀♀, Woodbine Station via Prairie (SAM AHC 31720, 31724, 32370); 13 ♂♂, 6 ♀♀, 17 km E of Jericho (SAM AHC 31587); 1 ♂, 5 ♀♀, 50 km W of Jericho (SAM AHC 31598); 1♀, 18 km W of Warwick (SAM AHC 25427); New South Wales: 11 ♂♂, 14 ♀♀, Rivertree (SAM AHC 6001, 22991);12 ♂♂, 10 ♀♀ Wollomombi (SAM AHC 44350).

***Representative DNA sequences*:** Molecular voucher from *O. robustus* (74 km W of Cloncurry; SAM48921): GenBank: MT080016 (ITS1, *5.8S* and ITS2).

**Description**

***General*.** Robust, whitish nematodes. Cephalic collar absent; mouth dorso-ventrally elongate; elevation on each side of mouth opening bears lateral amphid and 2 dome-shaped sub-median papillae; papillae with 2 short, anteriorly-directed setae. Buccal capsule elongate, dorso-ventrally elongate, poorly sclerotised, with partially sclerotised annulus in mid-region of buccal capsule; buccal capsule supported externally by strong radial musculature in posterior half; non- sclerotised annulus at junction of buccal capsule with oesophagus. Oesophagus elongate; corpus cylindrical, with *c.*35 sets of transverse sclerotised structures in lining, one arising from each sector of oesophagus (1 dorsal and 2 sub-ventral); isthmus short, slightly swollen; bulb elongate; intestinal cells enlarged at anterior extremity, surrounding posterior part of oesophageal bulb. Nerve-ring in anterior oesophageal region. Excretory pore at level of or posterior to oesophageal bulb; deirids at level of buccal capsule.

**Figs. 15–22:**
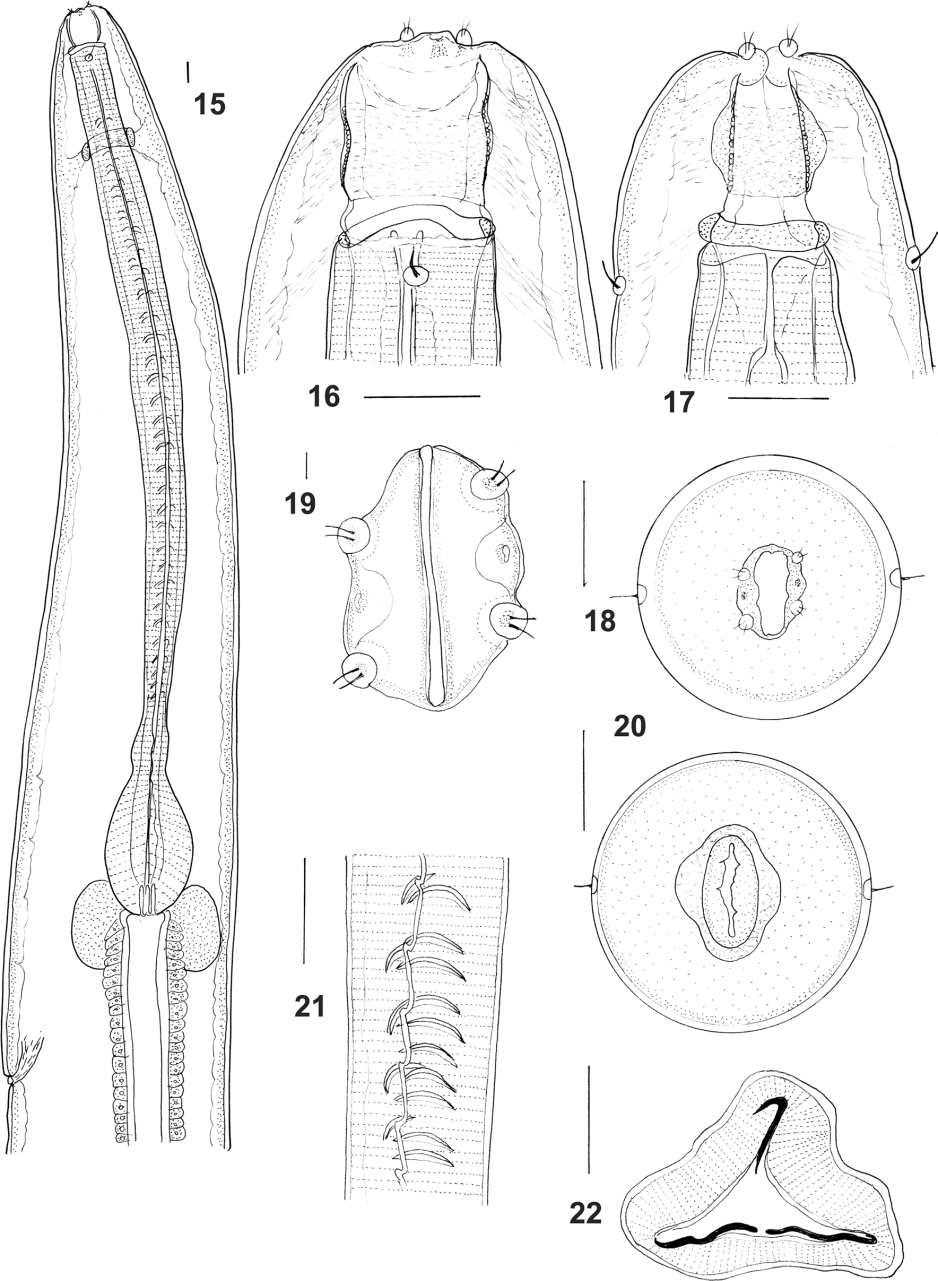
*Macroponema beveridgei* Mawson, 1978 from *Osphranter robustus* and *O. antilopinus*. **15** Anterior region, left lateral view. **16** Buccal capsule, lateral view. **17** Buccal capsule, ventral view. **18** Anterior extremity, apical view. **19** Mouth opening, apical view, showing detail of cephalic papillae and amphids. **20** Transverse optical section through buccal capsule. **21** Oesophageal corpus, showing transverse sclerotisations of lining. **22** Transverse section of oesophagus showing sclerotisations of lining. *Scale-bars*: **15**–**18**, **20**–**22**, 0.1 mm; **19**, 0.01 mm

**Figs. 23–27:**
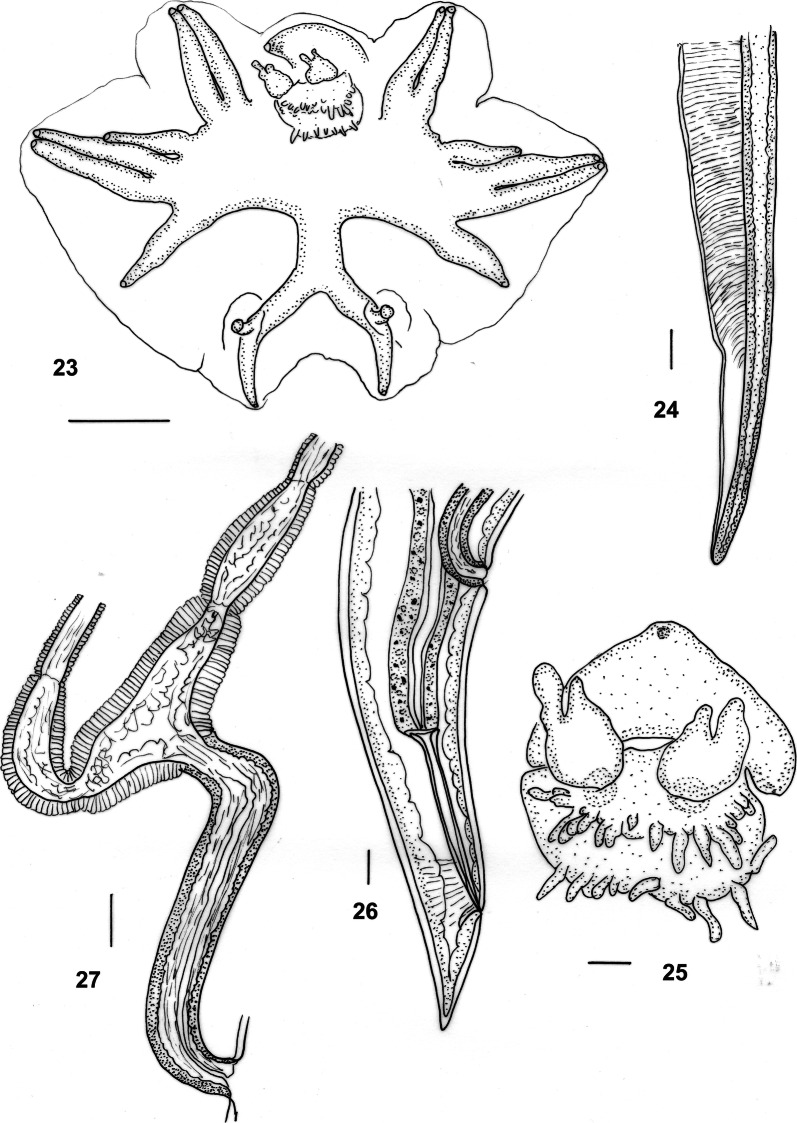
*Macroponema beveridgei* Mawson, 1978 from *Osphranter robustus* and *O. antilopinus*. **23** Bursa, apical view. **24** Spicule tip, left lateral view. **25** Genital cone, apical view. **26** Female tail, right lateral view. **27** Vagina and ovejector, right lateral view. *Scale-bars*: **23**, **26**, **27**, 0.1 mm; **24**, **25**, 0.01 mm

***Male*** [Measurements of 10 specimens; Figs. 15–25.] Total length 15.0–20.0 (18.2); maximum width 0.63–0.94 (0.75); buccal capsule 0.13–0.18 (0.14) long, 0.10–0.12 (0.11) wide; oesophagus 3.35–4.45 (4.00); nerve-ring from anterior extremity 0.65–0.92 (0.73); excretory pore from anterior extremity 3.88–5.00 (4.33); deirid from anterior extremity 0.13–0.22 (0.18). Bursal lobes poorly separated; lateral lobes slightly longer than ventral and dorsal lobes; dorsal lobe with median indentation; ventro-ventral and ventro-lateral rays apposed, reach margin of bursa; externo-lateral ray divergent from lateral trunk, not reaching margin of bursa; medio-lateral and postero-lateral rays apposed, reaching margin of bursa; externo-dorsal ray arising from lateral trunk, stout, not reaching margin of bursa; dorsal ray slender at origin, divides at 1/3 length; branches arcuate, internal branchlets reaching margin of bursa; external branchlets very short, arise midway between principal bifurcation and tips of internal branchlets, terminate in elevations on internal surface of bursa. Spicules elongate, alate; alae with numerous, fine, transverse striations; anterior extremity irregularly knobbed; distal extremity blunt tipped; alae diminish in width gradually anterior to spicule tip, lose striations prior to termination; spicule length 1.77–2.12 (1.95); gubernaculum absent; central cordate and paired lateral thickenings of spicule sheaths present. Ventral lip of genital cone large, conical, bearing papilla 0; dorsal lip with paired bifid appendages and 2 semi-circles of digitiform projections of varying lengths.

***Female*** [Measurements of 10 specimens; Figs. 26, 27.] Total length 40.0–62.0 (52.6); maximum width 0.85–1.15 (1.00); buccal capsule 0.13–0.22 (0.17) long, 0.12–0.17 (0.14) wide; oesophagus 4.38–5.33 (4.90); nerve-ring from anterior extremity 0.70–0.90 (0.81); excretory pore from anterior extremity 4.50–5.95 (5.35); deirid from anterior extremity 0.14–0.26 (0.19). Tail short, conical, 0.32–0.53 (0.45) long, deviated slightly dorsally; vulva 1.24–1.95 (1.52) from tip of tail; vagina short, 0.70–1.05 (0.84) long, slightly sinuous; egg ellipsoidal 0.10–0.13 (0.12) long, 0.04–0.06 (0.05) wide.

**Remarks**

The present re-description of *M. beveridgei* confirms that of Mawson [1] and provides additional details. The species can be readily distinguished from all congeners by its oesophageal ornamentation, with multiple transverse sclerotisations of the oesophageal lining.

Mawson [1] nominated *Osphranter antilopinus* (syn. *Macropus antilopinus*) as the type-host and Elizabeth Downs Station as the type-locality. It seems likely therefore that the specimens deposited as SAM AHC 6083 from the same host and locality represent paratypes, even though they were not designated as such in the original description. Mawson [1] also reported the species from *Notamacropus agilis* (syn. *Macropus agilis*) from Marrakai Plains (as Merridai Plains), but this appears to be an error. The material from this locality in SAM (SAM AHC 22992) is labelled as being from *O. antilopinus*. The records of this species occurring in *N. agilis* in Speare et al. [16] and in Spratt & Beveridge [17], based on the report by Mawson [1], are therefore suspect, with no supporting material from a wide range of collections from the same host species [16]. A further record of this species from *Wallabia bicolor* on Stradbroke Island, Queensland in Mawson [1] was again not substantiated based on collections in SAM and was not included in the parasites of this species of wallaby by Beveridge [18]. The validity of this record consequently needs to be confirmed.

In the present collections, *M. beveridgei* was most commonly encountered in *O. robustus*, probably because this species has been more intensely sampled than *O. antilopinus*. The molecular data suggest that nematodes from these two closely related macropodid species are identical. Also included in the molecular component of this study were specimens from *Notamacropus dorsalis* (Gray). This association was not reported by Beveridge et al. [19] based on an examination of 39 *N. dorsalis* from Queensland. The host from which the molecular specimens were obtained was collected at the same time as a specimen of *O. robustus* at the same locality, and while specimens were frozen for molecular studies, it appears that none were preserved for morphological study. Assuming that there were no errors in the labelling of specimens at the time of collection, this is the first record of *M. beveridgei* in *N. dorsalis*, but it requires confirmation.

**Fig. 28:**
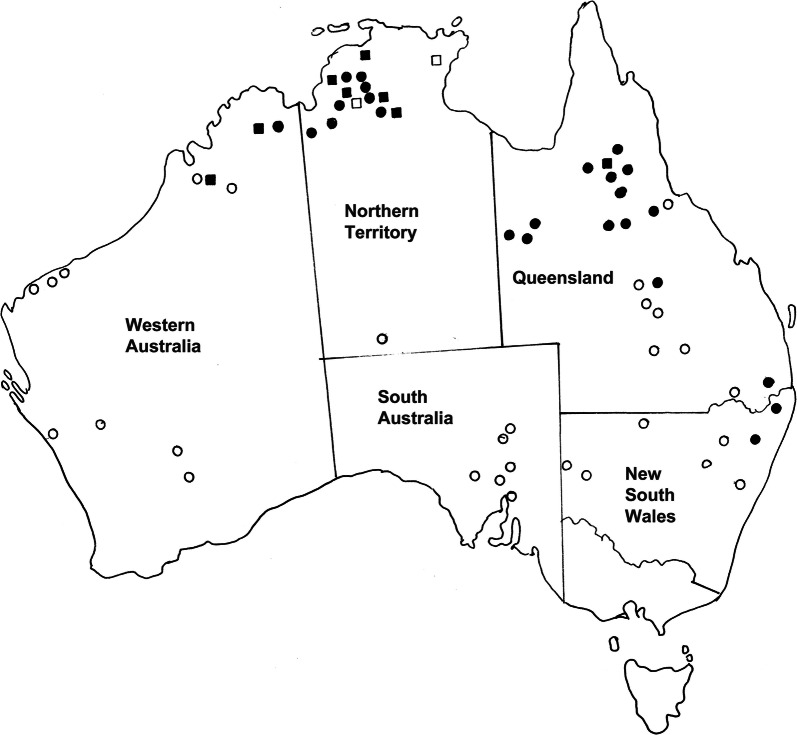
Distribution of *Macroponema beveridgei* Mawson, 1978 in *Osphranter robustus* and *O. antilopinus* in northern and eastern Australia. Closed circles represent localities at which the parasite has been collected from *O. robustus*; open circles indicate locations at which common wallaroos have been examined but the nematode has not been found; closed squares represent localities at which the parasite has been collected from *O. antilopinus*; open squares indicate locations at which antilopine wallaroos have been examined but the nematode has not been found

As noted by Beveridge [20], *M. beveridgei* has an unusual distribution (Fig. 28), occurring across northern Australia and along the east coast as far south as north-eastern New South Wales.

***Macroponema comani*Mawson, 1978**

***Type-host*:***Macropus giganteus* Shaw (Marsupialia: Macropodidae).

***Type-locality*:** Yan Yean (37°34′S, 145°06′E), Victoria, Australia.

***Type-material*:** Holotype ♂ (SAM AHC 41156); allotype ♀ (SAM AHC 41157).

***Additional material examined*:** Queensland: 2 ♀♀, 53 km N of The Lynd Junction (SAM AHC 48882); 1 ♀, 29 km N of The Lynd Junction (SAM AHC 48877); 3 ♂♂, 13 ♀♀, Hervey’s Range, Townsville (SAM AHC 6393); 3 ♂♂, 15 ♀♀, Oak Valley, Townsville (SAM AHC 24250, 32767); 13 ♀♀, Townsville (SAM AHC 7640); 3 ♂♂, 2 ♀♀, Woodstock (SAM AHC 7641); 3 ♂♂, 5 ♀♀, Landsdown Station via Woodstock (SAM AHC 7672); 10 ♀♀, 5 km S of Reid River (SAM AHC 32759); 2 ♂♂, 2 ♀♀, Mingela (SAM AHC 7396, 7826); 1♂, 3 ♀♀, Jumba Station via Charters Towers (SAM AHC 48879); 13 ♂♂, 30 ♀♀, Pallamana Station via Charters Towers (SAM AHC 13354, 13416); 31 ♂♂, 65 ♀♀, Harvest Home Station via Charters Towers (SAM AHC 13384, 13385, 13383, 13427); 1 ♂, Inkerman Station via Home Hill (SAM AHC 7658); 1 ♂, 1 ♀, Homestead (SAM AHC 32564); 11 ♂♂, 25 ♀♀, 6 km W of Prairie (SAM AHC 31715); 1 ♂, 1 ♀, 20 km N of Clermont (SAM AHC 48875); 1 ♂, 1 ♀, 14 km N of Clermont (50E); 1 ♂, 20 km S of Barcaldine (SAM AHC 31624); 1 ♂,1 ♀, Bogantungen (SAM AHC 24265); 8 ♂♂, 2 ♀♀, 66 km N of Rockhampton (SAM AHC 12132); 2 ♂♂, 1 ♀, 40 km N of Rockhampton (SAM AHC 11091); 2 ♂♂, 3 ♀♀, Rockhampton (SAM AHC 11061); 2 ♂♂, 2 ♀♀, Mount Hay via Rockhampton (SAM AHC 32479); 1 ♂, Darling Plains Station via Banana (SAM AHC 19905); 3 ♂♂, 3 ♀♀, 15 km S of Wowan (SAM AHC 48876); 1 ♂, 2 ♀♀, 6 km W of Warwick (SAM AHC 48880); 2 ♂♂, 2 ♀♀, 5 km E of Oman Ama (SAM AHC 48878); 2 ♂♂,1 ♀, Killarney (SAM AHC 19908); New South Wales: 1 ♂, 3 ♀♀, Ebor (SAM AHC 11073); 4 ♂♂, 12 ♀♀, Kingstown (SAM AHC 11074); Australian Capital Territory: 1 ♀, Tidbinbillla (SAM AHC 10942); Victoria: 6 ♂♂, 68 ♀♀, Yan Yean (SAM AHC 9593, 9595, 9597, 9600, 9625, 9622, 9626, 9627, 9661,11208); 7 ♂♂, 8 ♀♀, Dartmouth (SAM AHC 9218); 1 ♀, Bellbird (SAM AHC 9727); 1 ♀, Mitta Mitta (SAM AHC 12054); 9 ♂♂, 21♀♀, Marlo (SAM AHC 23001).

***Representative DNA sequences*:** Molecular voucher from *M. giganteus* (50 km N of The Lynd Junction; SAM48882): GenBank: MT080023 (ITS1, *5.8S* and ITS2).

**Description**

***General*.** Robust, whitish nematodes. Cephalic collar absent; mouth dorso-ventrally elongate; elevation on each side of mouth opening bears lateral amphid and 2 dome-shaped sub-median papillae; papillae with 2 short, anteriorly-directed setae. Buccal capsule elongate, dorso-ventrally elongate, poorly sclerotised, with partially sclerotised annulus in anterior half; shelf projects into buccal capsule at anterior extremity of buccal capsule, not visible in all specimens; buccal capsule supported externally by strong radial musculature in posterior half; sclerotised annulus at junction of buccal capsule with oesophagus; oesophagus elongate; corpus widening posterior to nerve-ring; posterior half of corpus with *c.*20 sets of bead-like sclerotised projections in lining, one arising from each sector of oesophagus; isthmus narrow, elongate; bulb elongate; intestinal cells enlarged at anterior extremity, surrounding posterior part of oesophageal bulb. Nerve-ring in anterior oesophageal region; excretory pore at level of oesophageal bulb; deirids at level of buccal capsule.

**Figs. 29–37:**
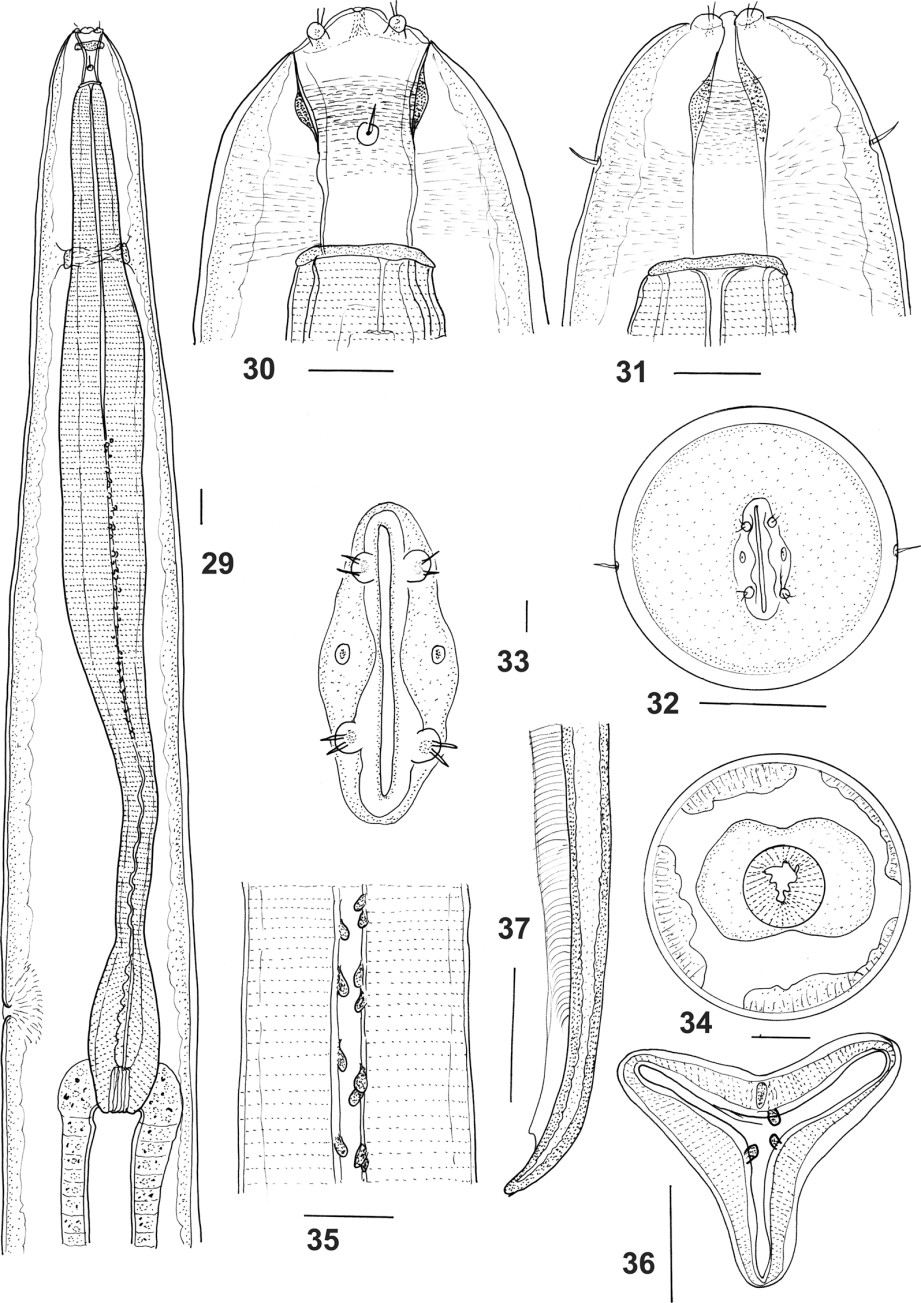
*Macroponema comani* Mawson, 1978 from *Macropus giganteus*. **29** Anterior region, left lateral view. **30** Buccal capsule, lateral view. **31** Buccal capsule, ventral view. **32** Anterior extremity, apical view. **33** Mouth opening, apical view, showing detail of cephalic papillae and amphids. **34** Transverse optical section through oesophagus at level of oesophageal bulb, showing anterior extension of intestine. **35** Oesophageal corpus, showing bead-like sclerotisations of lining. **36** Transverse section of oesophageal corpus, showing bead-like sclerotisations of lining. **37** Spicule tip, left lateral view. *Scale-bars*: **29**–**32**, **34**–**37**, 0.1 mm; **33**, 0.01 mm

**Figs. 38–44:**
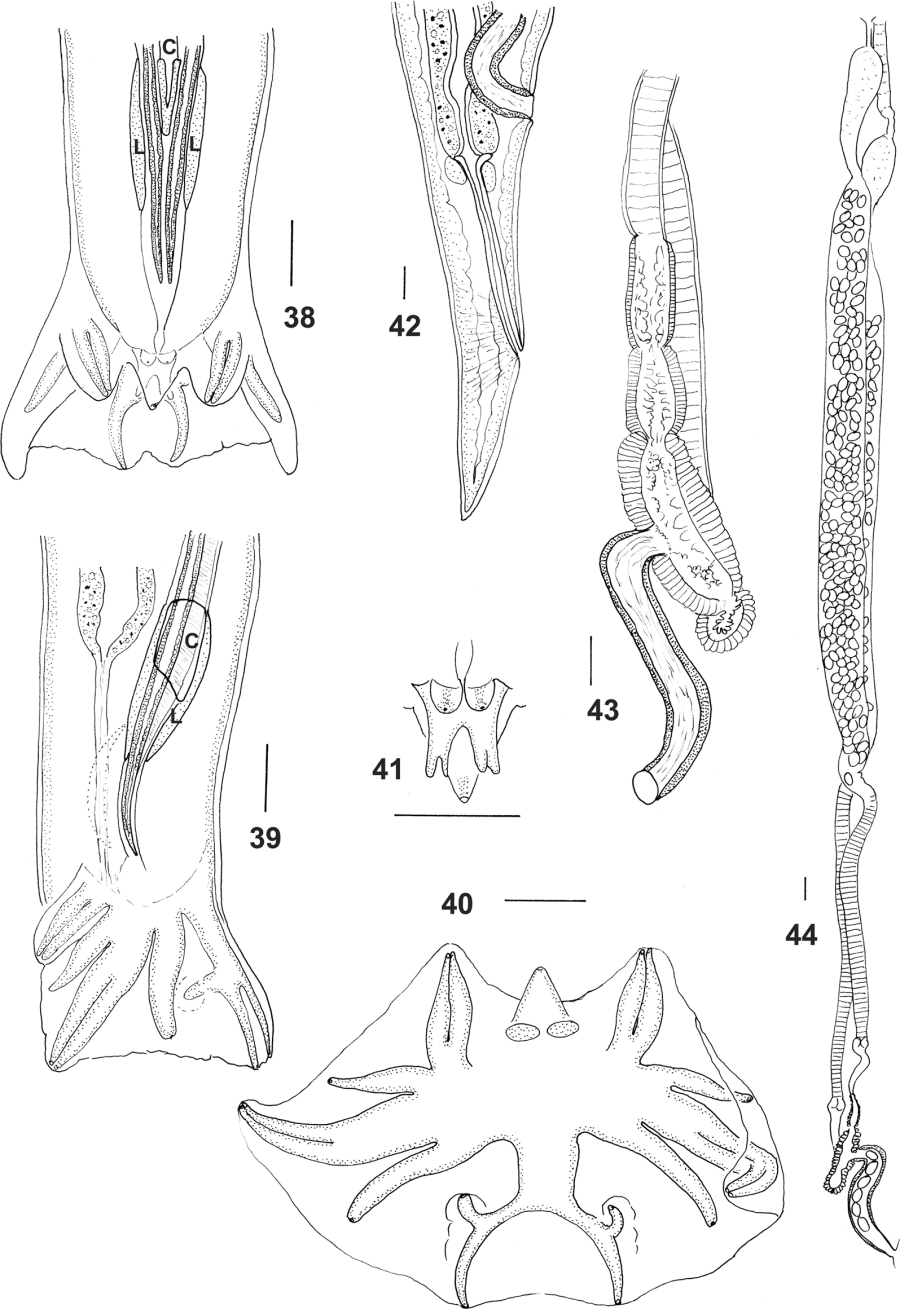
*Macroponema comani* Mawson, 1978 from *Macropus giganteus*. **38** Posterior end of male, ventral view, showing lateral (L) and central cordate (C) thickenings of spicule sheaths. **39** Posterior end of male, left lateral view, showing thickenings of spicule sheaths. **40** Bursa, apical view. **41** Genital cone, dorsal view. **42** Female tail, right lateral view. **43** Vagina and ovejector, left, lateral view. **44** Female genital system, showing uteri and seminal receptacles. *Scale-bars*: 0.1 mm

***Male*** [Measurements of 10 specimens; Figs. 29–41.] Total length 12.0–17.0 (14.1); maximum width 0.45–0.68 (0.58); buccal capsule 0.10–0.12 (0.11) long, 0.06–0.09 (0.08) wide; oesophagus 2.40–3.05 (2.65); nerve-ring from anterior extremity 0.58–0.72 (0.68); excretory pore from anterior extremity 2.28–3.68 (2.76); deirid from anterior extremity 0.12–0.15 (0.13). Bursal lobes poorly separated; lateral lobes slightly longer than ventral and dorsal lobes; no median indentation in dorsal lobe; ventro-ventral and ventro-lateral rays apposed, reach margin of bursa; externo-lateral ray divergent from lateral trunk, not reaching margin of bursa; medio-lateral and postero-lateral rays apposed in distal half, fused in proximal half, reaching margin of bursa; externo-dorsal ray arising from lateral trunk, stout, not reaching margin of bursa; dorsal ray slender at origin, divides at mid-length; branches arcuate, internal branchlets reaching margin of bursa; external branchlets very short, arise soon after principal bifurcation, terminate in elevations on internal surface of bursa. Spicules elongate, alate; alae with numerous, fine, transverse striations; anterior extremity irregularly knobbed; distal extremity blunt-tipped; alae terminate abruptly anterior to spicule tip, lose striations prior to termination; spicule length 1.70–1.98 (1.81); gubernaculum absent; central cordate and paired lateral thickenings of spicule sheaths present. Ventral lip of genital cone large, conical, bearing papilla 0; dorsal lip with paired bifid appendages.

***Female*** [Measurements of 10 specimens. Figs. 42–44.] Total length 18.0–27.0 (21.1); maximum width 0.67–0.94 (0.85); buccal capsule 0.12–0.16 (0.14) long, 0.08–0.11 (0.09) wide; oesophagus 2.95–4.32 (3.36); nerve-ring from anterior extremity 0.70–0.87 (0.80); excretory pore from anterior extremity 2.88–4.05 (3.50); deirid from anterior extremity 0.13–0.21 (015). Tail short, conical, straight, 0.41–0.60 (0.48) long; vulva 0.85–1.45 (1.29) from tip of tail; vagina short, 0.55–0.95 (0.78) long, slightly sinuous; distal uterus narrow, enlarging and becoming filled with eggs proximally; proximal tip of uterus bulbiform (seminal receptacle), with narrow duct leading to ovary; egg ellipsoidal 0.10–0.12 (0.11) long, 0.06–0.07 (0.06) wide.

**Remarks**

The present redescription confirms that of Mawson [1] but provides additional details including egg sizes and a line drawing of the genital cone, which was illustrated by a scanning electron micrograph only by Mawson [1]. The transverse section of the oesophagus illustrated by Mawson [1] (figure 16 in [1]) suggests that the bead-like thickenings lining the oesophagus are arranged in pairs on each segment (dorsal and two subventral) of the oesophagus. This is not the case (Fig. 36) and a single structure is present for each segment of the oesophagus at each level. Mawson [1] commented on the plasticity of the buccal capsule providing ventral and sublateral views with the buccal capsule in collapsed and expanded positions. Similar variation was observed in the specimens here, but the illustrations presented in the redescription are limited to a ventral view with the buccal capsule collapsed. Mawson [1] did not illustrate or describe the complex array of muscles running between the wall of the buccal capsule and the longitudinal musculature shown here. They are similar in their arrangement to those described by Beveridge [21]. The illustrations of Beveridge [21] complement those presented here and have not been included in the present redescription along with the projections into the lumen of the buccal capsule at its anterior extremity. Beveridge [21] considered the host of his specimens of *M. comani* to be *O. robustus* which he noted was unusual. However, examining the range of nematodes collected from this individual host and the fact that it was collected at the same time as the material from *Ma. giganteus*, it seems most likely that the original host identification was in error and consequently the material (SAM AHC 13354) has been included above under *Ma. giganteus*.

Although a wide range of specimens was available, very few were gravid, probably accounting for the lack of any description of the egg by Mawson [1]. However, in some of the new specimens, the entire female reproductive tract was visible (Fig. 44) and indicated that unlike in species of other genera of the Cloacininae, the distal uterus is narrow and tubular, eventually becoming saccate anteriorly, with eggs largely restricted to the saccate region. In addition, a seminal receptacle is detectable at the proximal extremity of the uterus (Fig. 44). The central cordate and paired lateral thickenings of the spicule sheaths characteristic of the family [22], have not been reported previously for this genus, but are here illustrated in Figs. 38 and 39.

**Fig. 45:**
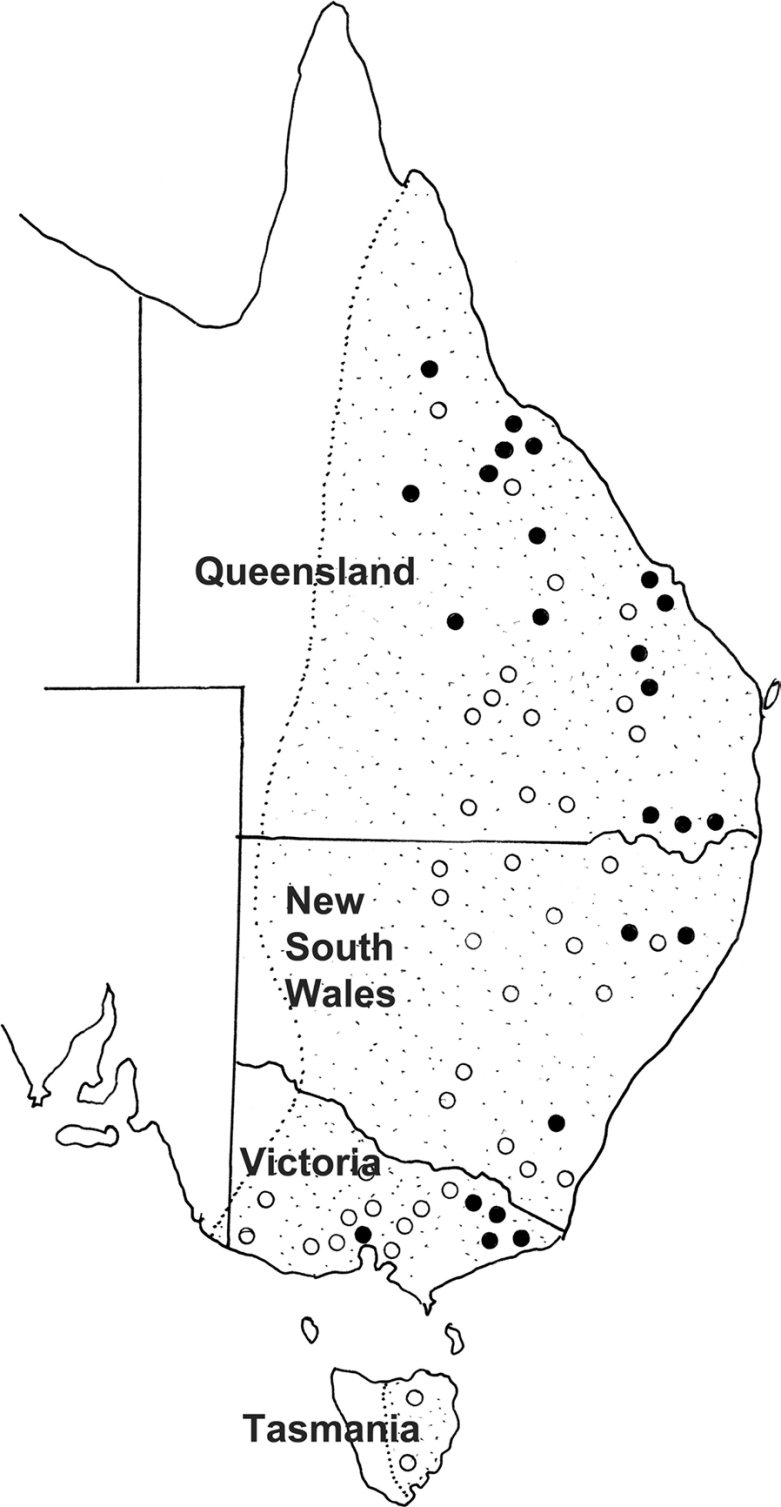
Distribution of *Macroponema comani* Mawson, 1978 from *Macropus giganteus* in eastern Australia. Closed circles represent localities at which the parasite has been collected; open circles indicate locations at which kangaroos have been examined but the nematode has not been found. The stippled area indicates the known geographical range of *M. giganteus*

The current data confirm the restriction of this species to a single host, *Ma. giganteus*, but indicates that its distribution extends from Victoria to northern Queensland (Fig. 45).

***Macroponema obendorfi*Beveridge n. sp.**

Syn. *Macroponema* cf. *comani* of Tan et al. (2012)

***Type-host*:***Osphranter robustus woodwardi* (Thomas) (Marsupialia: Macropodidae).

***Additional host:****Osphranter antilopinus* (Gould) (Marsupialia: Macropodidae).

***Type-locality*:** Mount Smith (13°31′S, 131°7′E), Northern Territory, Australia.

***Type-material*:** Holotype ♂ (SAM AHC 48936); allotype ♀ (SAM AHC 48937); 16 paratypes: 7♂♂ and 9 ♀♀ (SAM AHC 48938).

***Additional material examined*:** From *O. robustus woodwardi*: Northern Territory: 1♂, 4♀♀, Katherine (SAM AHC 32700, 48883); 2 ♂♂, 4 ♀♀, Newry Station via Timber Creek (SAM AHC 44339). From *O. antilopinus*: Northern Territory: 1♂, 1♀, Katherine (SAM AHC 32710); Western Australia: 5 ♀♀, Napier Downs Station via Derby (SAM AHC 48884); 2 ♀♀, Camp Creek, Mitchell Plateau (SAM AHC 6162).

***Representative DNA sequences*:** Molecular voucher from *O. robustus* (Newry Stn via Timber Creek; SAM46097): GenBank: HE775532 (ITS1, *5.8S* and ITS2).

***Etymology*:** Named after Dr D. L. Obendorf, who helped collect much of the original material upon which the description of *M. beveridgei* and the genus was based.

**Description**

***General*.** Robust, whitish nematodes; cephalic collar absent; mouth dorso-ventrally elongate; elevation on each side of mouth opening bears lateral amphid and 2 dome-shaped sub-median papillae; papillae with 2 short, anteriorly-directed setae; buccal capsule elongate, dorso-ventrally elongate, poorly sclerotised, with partially sclerotised annulus in anterior half; buccal capsule supported externally by strong radial musculature in posterior half; non-sclerotised annulus at junction of buccal capsule with oesophagus; oesophagus elongate; corpus widening posterior to nerve-ring; posterior half of corpus with *c*.12–14 sets of bead-like sclerotised projections in lining, one arising from each sector of oesophagus; isthmus narrow, elongate; bulb elongate; intestinal cells enlarged at anterior extremity, surrounding posterior extremity of oesophageal bulb. Nerve-ring in anterior oesophageal region; excretory pore at level of oesophageal bulb or anterior to it; deirids at level of buccal capsule.

**Figs. 46–54:**
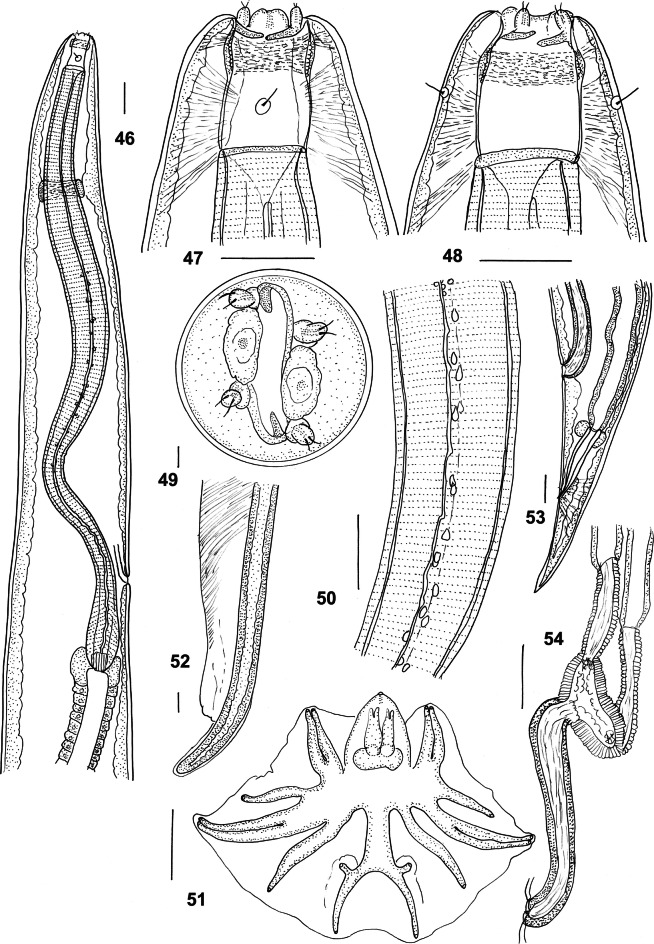
*Macroponema obendorfi* n. sp. from *Osphranter robustus* and *O. antilopinus*. **46** Anterior region, right lateral view. **47** Buccal capsule, lateral view. **48** Buccal capsule, ventral view. **49** Anterior extremity, apical view. **50** Oesophageal corpus, showing bead-like sclerotisations of lining. **51** Bursa, apical view. **52** Spicule tip, left lateral view. **53**, Female tail, left lateral view. **54** Vagina and ovejector, left lateral view. *Scale-bars*: **46**–**48, 50**, **51, 53**–**54,** 0.1 mm; **49**, **52**, 0.01 mm

***Male*** [Measurements of 10 specimens; Figs. 46–52.] Total length 8.3–11.1 (10.3); maximum width 0.42–0.63 (0.50); buccal capsule 0.09–0.13 (0.12) long, 0.04–0.07 (0.06) wide; oesophagus 2.45–2.72 (2.52); nerve-ring from anterior extremity 0.45–0.63 (0.58); excretory pore from anterior extremity 1.98–2.45 (2.28); deirid from anterior extremity 0.08–0.14 (0.11). Bursal lobes poorly separated; lateral lobes slightly longer than ventral and dorsal lobes; no indentation in dorsal lobe; ventro-ventral and ventro-lateral rays apposed, reaching margin of bursa; externo-lateral ray divergent from lateral trunk, not reaching margin of bursa; medio-lateral and postero-lateral rays apposed in distal half, fused in proximal half, reaching margin of bursa; externo-dorsal ray arising from lateral trunk, slender, not reaching margin of bursa; dorsal ray slender at origin, divides at mid-length; branches arcuate, internal branchlets reaching margin of bursa; external branchlets very short, arise soon after principal bifurcation, terminate in elevations on internal surface of bursa. Spicules elongate, alate; alae with numerous, fine, transverse striations; anterior extremity irregularly knobbed; distal extremity blunt tipped; alae terminate abruptly anterior to spicule tip, lose striations prior to termination; spicule length 1.43–1.80 (1.66); gubernaculum absent; central cordate and paired lateral thickenings of spicule sheaths present. Ventral lip of genital cone large, conical, bearing papilla 0; dorsal lip with paired bifid appendages.

***Female*** [Measurements of 10 specimens; Figs. 53, 54.] Total length 11.3–13.8 (13.1); maximum width 0.69–0.80 (0.74); buccal capsule 0.10–0.15 (0.12) long, 0.05–0.07 (0.06) wide; oesophagus 2.60–3.36 (2.94); nerve-ring from anterior extremity 0.62–0.73 (0.67); excretory pore from anterior extremity 2.15–3.30 (2.72); deirid from anterior extremity 0.08–0.17 (013). Tail short, conical, straight, 0.35–0.48 (0.42) long; vulva 0.90–1.41 (1.02) from tip of tail; vagina short, 0.45–0.73 (0.65) long, slightly sinuous; eggs not seen.

**Remarks**

**Fig. 55:**
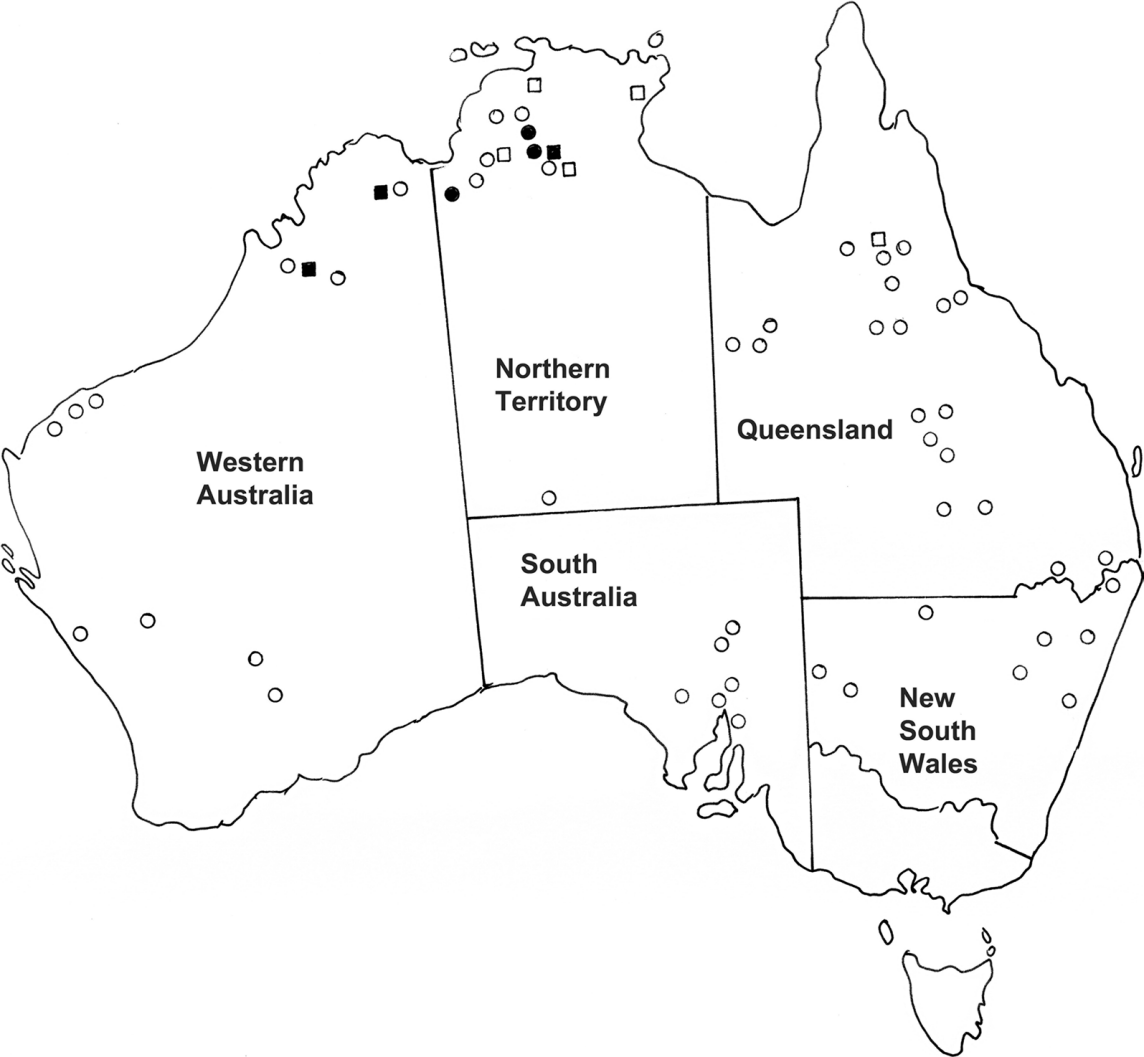
Distribution of *Macroponema obendorfi* n. sp. in *Osphranter robustus* and *O. antilopinus* in northern Australia. Closed circles represent localities at which the parasite has been collected from *O. robustus*; open circles indicate locations at which common wallaroos have been examined but the nematode has not been found; closed squares represent localities at which the parasite has been collected from *O. antilopinus*; open squares indicate locations at which antilopine wallaroos have been examined but the nematode has not been found

This species was initially identified as *M*. cf. *comani* by Tan et al. [4] based on molecular differences and a difference in host distribution with *M. comani* restricted to *Ma. giganteus* and *M*. cf. *comani* occurring in *O. r. woodwardi.* The metrical morphological data included in that study [4] indicated no obvious differences between the populations of *M. comani* distinguishable using molecular methods. In the present morphological study, a few features were identified to separate *M. comani* occurring in *Ma. giganteus* from the closely related species, here identified as *M. obendorfi* n. sp. occurring in *O. robustus*. The simplest feature to observe is the number of groups of bead-like sclerotised projections in the oesophagus which range from 12 to 14 in *M. obendorfi* n. sp. compared with 20 in *M. comani*. There are slight differences in the mean spicule length (1.66 mm in *M. obendorfi* n. sp. vs 1.81 mm in M. *comani*) but there is considerable overlap. The two species also share the unusual feature of having the medio-lateral and postero-lateral rays fused in the proximal region and apposed only in their distal regions, in contradistinction to the two rays being apposed along their entire lengths in the remaining species. The two species appear to be very similar morphologically with no additional differences noted apart from the numbers of groups of sclerotised beads lining the oesophagus and the anomalous host and geographical distribution (Fig. 55).

## Discussion

The present review of the genus *Macroponema* using both molecular and morphological approaches has established the presence of four species within the genus. The previously identified species, *M. beveridgei* and *M. comani*, are re-described and their host and geographical ranges defined in greater detail. *Macroponema* cf. *comani* of Tan et al. [4] was confirmed as a distinct species and, with its description as *M. obendorfi* n. sp., its differential morphological features were defined for the first time. In addition, a fourth species, *M. arundeli* n. sp. was identified in *Ma. giganteus*, and has been characterised using both morphological and molecular methods.

While the four species are most easily identified based on the ornamentation of the oesophagus, in poorly preserved specimens, this may be difficult to discern. The females of *M. arundeli* n. sp. and *M. beveridgei* are significantly larger (37–70 and 40–62 mm, respectively) than their synhospitalic species *M. comani* and M. *obendorfi* n. sp. (18–27 and 11–13 mm, respectively). In addition, their tails differ with those of *M. arundeli* n. sp. and *M. beveridgei* being deviated slightly dorsally (Figs. 12 and 26), while those of *M. comani* and *M. obendorfi* n. sp. are directed posteriorly (Figs. 42 and 53). Size differences in the synhospitalic males are less marked but exhibit a similar pattern with those of *M. arundeli* n. sp. and *M. beveridgei* being significantly larger (19–25 and 15–20 mm, respectively) than their synhospitalic species *M. comani* and M. *obendorfi* n. sp. (12–17 and 8–11 mm, respectively). The spicule tips of *M. arundeli* n. sp. and *M. beveridgei* differ from those of *M. comani* and M. *obendorfi* n. sp. in that the ala diminishes in width towards the tip of the spicule in the former pair of species (Figs. 10, 24) while in the latter pair, the ala terminates abruptly anterior to the spicule tip (Figs. 37, 52). The genital cone of *M. beveridgei* differs from all congeners in having multiple appendages surrounding the dorsal lip (Fig. 25).

Three of the species appear to be highly host-specific with *M. arundeli* n. sp. and *M. comani* present only in *Ma. giganteus* and *M. obendorfi* n. sp. found only in *O. robustus woodwardi*. There is a single report of *M. comani* from *O. robustus robustus* in north-eastern Queensland [21], but other associated nematodes suggest that this is a case of host misidentification, particularly since specimens of *Ma. giganteus* were collected at the same locality on the same date and consequently, the host associations of this collection have been changed.

*Macroponema beveridgei* is primarily a parasite of *O. robustus*, and although found in all three subspecies, *O. r. robustus* (eastern New South Wales and Queensland), *O. r. erubescens* (north-western Queensland) and *O. r. woodwardi* (Northern Territory and north-western Western Australia), appears to be limited to the east and north of the continent (Fig. 28). It has also been found in the closely related and sympatric species, *O. antilopinus* (the type-host), although this is not surprising as *O. robustus* and *O. antilopinus* share approximately 55% of their helminth species in northern Queensland [19]. *Notamacropus dorsalis* was reported for the first time as a host of *M. beveridgei* by Chilton et al. [23], but the record requires confirmation as a previous survey of the helminths of this host species did not include this nematode species [19].

The generic independence of *Macroponema* within the tribe Macropostrongylinea is strongly supported by molecular studies, including *M. beveridgei* and *M. comani*, by Chilton et al. [23]. Morphologically, its key distinguishing features are the dorso-ventrally elongated mouth opening, the laterally compressed buccal capsule (Figs. 5, 18, 32, 49) and the ornamentation of the lining of the oesophagus. Mawson [1] utilised a different series of morphological characters apart from the laterally compressed buccal capsule which she considered to occur also in *Macropostrongylus* Yorke & Maplestone, 1926 and *Papillostrongylus* Johnston & Mawson, 1939. She noted differences in the ventral lobes of the bursa which she considered to be joined in *Macropostrongylus* and *Popovastrongylus* Mawson, 1977, while separate in *Macroponema* and *Papillostrongylus*, as well as *Macropostrongylus* having “longitudinal ridges” in the buccal capsule which were absent in *Macroponema* [1]. The present descriptions and redescriptions of species of *Macroponema* suggest that the ventral lobes are joined ventrally in all species, thereby invalidating this feature as a generic character. This feature is not clearly visible unless apical views of the bursa are illustrated, and such views are presented here for the first time. The buccal capsule is not laterally compressed in all species of *Macropostrongylus* [24] and the “longitudinal ridges” of the buccal capsule of *Macropostrongylus* are present in the type-species, *M. macropostrongylus* Yorke & Malpestone, 1926 (see figure 1F in [24]), in *M. yorkei* Baylis, 1927 (see figure 3E in [24]) and in *M. macrostoma* Davey & Wood, 1938 (see figure 5H in [24]), but not in the remaining species [24]. Consequently, this character should not be used in the discrimination of the two genera. In summary, the lateral compression of the mouth opening and the oesophageal ornamentation appear to be the two defining morphological characteristics of the genus.

Few of the female nematodes reported in this study were gravid, such that details of the eggs were not available for *M. arundeli* n. sp. and *M. obendorfi* n. sp. This may be due to the seasonal development of the nematodes. In the related genera *Labiosimplex* Smales, 1995 and *Labiomultiplex* Smales, 1994, in species such as *Labiosimplex longispicularis* Wood, 1929 and *Labiomultiplex eugenii* Johnston & Mawson, 1940, gravid females are only present during the months of the year that survival of eggs and larval stages is likely to be optimum [25–27]. Similar phenomena may be operating in the case of species of *Macroponema*, but this remains to be investigated.

While the currently available phylogenetic data suggest that *M. obendorfi* n. sp. has arisen by a host switch from *Ma. giganteus* to *O. robustus*, the relationships of *M. arundeli* n. sp. and *M. comani* in *Ma. giganteus* remain unclear. However, the geographical distributions of these species (Figs. 14, 45), suggest that the evolution of the genus has occurred primarily in eastern and northern Australia. Beveridge [20] noted the unusual distribution of *M. beveridgei* in *O. robustus*, being absent in South and much of Western Australia. The currently available but limited data, therefore, suggest the evolution of *M. beveridgei* in *O. robustus* and *M. arundeli* n. sp. in *Ma. giganteus*, followed by the evolution of *M. comani* within the same host species and of *M. obendorfi* n. sp. by a host switch into *O. robustus*. These are clearly tentative hypotheses suggested by the available data which necessitate more rigorous testing.

## Conclusions

Based on morphological and molecular characterisation of nematodes, this study revealed the existence of at least four species within the genus *Macroponema*. The morphology of two previously described species, *M. beveridgei* and *M. comani*, was redescribed whereas *Macroponema* cf. *comani* of Tan et al. [4] was confirmed as a distinct species as *M. obendorfi* n. sp. In addition, a fourth species, *M. arundeli* n. sp. was described from *Ma. giganteus*. The current phylogenetic data suggest that *Macroponema* spp. plausibly evolved by host switching; however, further studies are required to test this hypothesis.

## Data Availability

All data generated or analysed during this study are included in this published article. The type material was deposited in the Australian Helminthological Collection, South Australian Museum. DNA sequence data generated during this study are available from the GenBank database under the accession nos. MT080008-MT080027.
